# Construction of a High-Density Genetic Linkage Map and QTL Mapping for Stem Rot Resistance in Passion Fruit (*Passiflora edulis* Sims)

**DOI:** 10.3390/genes16010096

**Published:** 2025-01-17

**Authors:** Yanyan Wu, Weihua Huang, Jieyun Liu, Junniu Zhou, Qinglan Tian, Xiuzhong Xia, Haifei Mou, Xinghai Yang

**Affiliations:** 1 Biotechnology Research Institute, Guangxi Academy of Agricultural Sciences, Nanning 530007, China; wuyanyan@gxaas.net (Y.W.); huangweihua@gxaas.net (W.H.); 1121.zjn@gxaas.net (J.Z.); tianqinglan@gxaas.net (Q.T.); mhf@gxaas.net (H.M.); 2 Guangxi Key Laboratory of Rice Genetics and Breeding, Rice Research Institute, Guangxi Academy of Agricultural Sciences, Nanning 530007, China; xiaxiuzhong@gxaas.net

**Keywords:** passion fruit, single nucleotide polymorphisms, genetic map, linkage analysis, stem rot resistance, candidate genes

## Abstract

Background: The passion fruit (*Passiflora edulis* Sims) is a diploid plant (2n = 2x = 18) and is a perennial scrambling vine in Southern China. However, the occurrence and spread of stem rot in passion fruit severely impact its yield and quality. Methods: In this study, we re-sequenced a BC_1_F_1_ population consisting of 158 individuals using whole-genome resequencing. We constructed a high-density genetic linkage map and identified the quantitative trait locus (QTL), and analyzed candidate genes associated with stem rot resistance in passion fruit. Results: Based on the passion fruit reference genome (MER), a high-density genetic linkage map was constructed with 1,180,406 single nucleotide polymorphisms (SNPs). The map contains nine linkage groups, covering a total genetic distance of 1559.03 cM, with an average genetic distance of 311.81 cM. The average genetic distance between 4206 bins was 0.404 cM, and the average gap length was 10.565 cM. The collinearity correlation coefficient between the genetic map and the passion fruit genome was 0.9994. *Fusarium solani* was used to infect the BC_1_F_1_ population, and the resistance to stem rot showed a continuous distribution. A QTL, *qPSR5*, was mapped to the 113,377,860 bp–114,811,870 bp genomic region on chromosome 5. We performed RNA sequencing (RNA-seq) and real-time quantitative polymerase chain reaction (RT-qPCR) to analyze the expression levels of predicted genes in the candidate region and identified *ZX.05G0020740* and *ZX.05G0020810* as ideal candidate genes for stem rot resistance in passion fruit. Conclusions: The findings in this study not only lay the foundation for cloning the *qPSR5* responsible for stem rot resistance but also provide genetic resources for the genetic improvement of passion fruit.

## 1. Introduction

The passion fruit (*Passiflora edulis* Sims) is an herbaceous vine plant, known for its aromatic fruit, which is rich in sugars, vitamins, and essential minerals such as calcium, iron, and zinc, offering high nutritional value. However, stem rot, caused by fungi such as *Fusarium oxysporum* [1], *Fusarium solani* [2], and *Phytophthora nicotianae* [3], has been spreading in many areas around the world [1,4,5,6]. This disease manifests itself as the initial symptoms of water-stained decay in the stem base. After being infected, the plants gradually wither, their leaves drop, and later the whole plant becomes yellow and withered to death, leading to the destruction of the garden [4,7], and this disease has caused serious losses to the growers.

In production, the prevention and control of passion fruit stem rot mainly rely on biological and chemical methods. Wang et al. identified *Bacillus velezensis* with a growth inhibitory effect against *Fusarium solani* [2]. Thuranira et al. proposed an integrated and sustainable approach towards management of passion fruit wilt disease by using a biocontrol agent (*Trichoderma asperellum*) and organic matter [8]. Chen et al. found that *Bacillus subtilis* 151B1 and YBC are potential biocontrol agents for passion fruit disease caused by *Fusarium solani* [9]. However, chemical and biological control, as the main methods of stem rot prevention and control, not only pollute the environment but also increase economic costs when used for a long time. Previous studies have shown that using molecular breeding techniques to develop disease-resistant varieties is the most effective and economical method for disease control [10]. Therefore, it is necessary to identify the resistance genes to stem rot disease in passion fruit [11], which has important theoretical and practical significance for the breeding of resistant varieties of passion fruit.

At present, there is limited study on the resistance genes to stem rot disease in passion fruit. Wu et al. analyzed 16 candidate genes for quantitative real-time PCR analysis in *Passiflora edulis* under stem rot conditions, and *C21209* and *EF-1α-2* were the most stable reference genes [4]. Using RNA-seq and weighted gene co-expression network analysis, Wu et al. found that genes related to lignin biosynthesis likely play critical roles in passion fruit stem rot resistance [12]. Cell wall lignification is a physical barrier for plants to resist pathogen invasion, and studies have shown that lignin is involved in stem rot resistance. Yang et al. found that *TaDIR-B1* may regulate wheat’s resistance to stem rot by altering lignin content [13]. Lv et al. cloned the gene *TaCWI*, which confers resistance to both wheat stem rot and sheath blight. The gene encodes a cell wall invertase protein that inhibits the expression of the *TaGAL* gene, counteracting its cell wall degradation effect and thickening the cell wall to resist pathogen invasion [14]. Chen et al. used transcriptome analysis to reveal that the increased resistance of hexaploid wheat to crown rot may be related to higher levels of phenylalanine ammonia-lyase (PAL) regulation of lignin and SA biosynthesis pathways [15]. Liu et al. validated that overexpression of *ZmBGLU17* does not affect the growth and yield of maize, but can significantly enhance its resistance to *Pythium* stalk rot [16]. Cao et al. found that phenolic compounds involved in lignin biosynthesis may slow down or inhibit the reproduction of maize stalk rot bacteria (*Fusarium verticillioides*) [17]. Cao et al. demonstrated that knocking out the *BnF5H* gene to reduce the S/G lignin ratio can enhance the stem rot resistance of *Brassica napus* [18].

Genetic maps are used for gene/QTL mapping to obtain genome fragments that are linked to the target trait [19,20]. In the genetic map studies of passion fruit, Carneiro et al. and Lopes et al. used 380 random amplified polymorphic DNA (RAPD) markers and 174 amplified fragment length polymorphism (AFLP) markers, respectively, to construct genetic maps for yellow passion fruit varieties IAPAR123 and IAPAR06, with both maps containing nine linkage groups. Later, Oliveira et al. reconstructed an integrated map for yellow passion fruit using 253 AFLP markers and 107 simple sequence repeat (SSR) markers, which contained 10 linkage groups. So far, although researchers have developed molecular genetic maps for passion fruit, these maps suffer from low marker density, limited genome coverage, and low detection efficiency, limiting their application. Therefore, to accelerate the molecular breeding process for disease resistance in cultivated passion fruit, it is essential to construct a high-density genetic map for mining stem rot resistance genes.

In a previous study, we found that Huangguoyuanshengzhong (HG) has good resistance to stem rot [4] and constructed a BC_1_F_1_ population. In order to explore the resistance genes to stem rot in passion fruit, we constructed a genetic map using resequencing technology and mapped a stem rot resistance QTL *qPSR5* on chromosome 5 in passion fruit. Candidate gene analysis revealed that *ZX.05G0020700*, *ZX.05G0020740*, and *ZX.05G0020810* may be involved in the resistance to stem rot in passion fruit. The study provides genetic resources and valuable information for the genetic improvement of stem rot disease resistance in passion fruit.

## 2. Materials and Methods

### 2.1. Characteristics of Passiflora edulis Sims

*Passiflora edulis* Sims, also known as passion fruit, is an herbaceous vine plant belonging to the *Passiflora* genus in the Passifloraceae family. It is native to the Americas and is widely planted in tropical and subtropical regions, including Guangxi, Guangdong, Yunnan, Fujian, and Guizhou in China. The most suitable growth temperature for passion fruit is 20–30 °C, and temperatures below 15 °C inhibit nutritional growth. All types of soil are suitable except for heavy clay, with a soil pH value of 5.5–6.5 being appropriate. The main artificial propagation methods for passion fruit (*Passiflora edulis* Sims) are sowing propagation, cutting propagation, or grafting propagation. Its unique flower shape and bright colors have high ornamental and greening value. The fruit has a spherical shape and is rich in vitamins and mineral elements. It can be eaten fresh and processed. The fruit juice has a strong aroma and contains nearly a hundred aroma components. It is known as the king of fruit juice and is commonly used in the processing of beverages such as fruit juice and fruit wine.

### 2.2. Plant Materials and Disease Incidence

In our previous study, we screened out the stem rot-resistant cultivar Huangguoyuanshengzhong (HG) [4]. Ziguo 7 (ZG7) originates from crossing Tainong 1 with Jinlingziguo; the offspring bred the excellent cultivar ZG 7, but this variety was not resistant to stem rot disease [12]. In 2018, an F_1_ generation was obtained by crossbreeding ZG7 (female parent) and HG (male parent). Then, we used the F_1_ generation to hybridize with ZG7 to obtain the BC_1_F_1_ population. In 2020, the experimental materials, HG, ZG7, and 158 progenies, were planted at the Meilinanfang site of the Guangxi Academy of Agricultural Sciences (108.16° E, 22.77° N). The total nitrogen content of the cultivated soil in the experimental site is 0.99 g·kg^−1^, total phosphorus is 0.85 g·kg^−1^, total potassium is 6.22 g·kg^−1^, organic matter is 17.71 g·kg^−1^, alkaline nitrogen is 81.04 mg·kg^−1^, available phosphorus is 56.16 mg·kg^−1^, available potassium is 157.29 mg·kg^−1^, and the soil pH is 6.06. Water and fertilizer management: 15 days before planting, apply 4–5 kg of organic fertilizer and 0.5 kg of calcium magnesium phosphate fertilizer as base fertilizer per planting hole. After 15 days of planting, apply drip irrigation with 15-15-15 water-soluble fertilizer at 15–20 g per plant, 3–5 times a month, and spray 0.3% potassium dihydrogen phosphate during the flowering and fruiting period. During the seedling stage, it is necessary to avoid flooding and root rot. Adequate water supply should be ensured during the flowering and fruit enlargement periods to promote nutrient absorption and fruit enlargement.

Stem rot samples were collected from the boundary between healthy and infected tissue at the stem of passion fruit plants, and the pathogen was isolated and placed on potato dextrose agar (PDA) medium, primarily identified as *Fusarium solani*. To assess the disease resistance of the passion fruit plants, we used an in vitro inoculation method. The purified and preserved pathogen was reactivated and cultured on PDA medium at 28 °C for 5 days. Mycelial plugs were obtained using a 1000 μL sterilized pipette tip. For the disease resistance evaluation, an artificial wound was made at the stem, and the prepared mycelial plugs were placed on the wound, with the mycelium facing directly against the wound site. Each BC_1_F_1_ individual was planted with 15 ramets, and the disease incidence rate was recorded. After 30 days of inoculation with *Fusarium solani*, we surveyed the resistance of each individual. If the site of the puncture was infected by the pathogen and turned brown or black-brown, or if the length of the lesion extended to more than 3 mm, or if the upper part of the stem died, it was considered a diseased plant. The calculation formula of incidence rate and susceptibility index is as follows: P = m/n × 100%, where P is the incidence rate, m is the number of diseased plants, and n is the total number of investigated plants.

### 2.3. DNA Extraction, SNP Detection, and SNP Genotyping

2021, at the 6–8 leaf stage, young leaves from the two parents and 158 individuals were collected and stored at −80 °C. Genomic DNA from the parents and BC_1_F_1_ population was extracted using the CTAB method [21]. The DNA concentration was measured with a Qubit 2.0 Fluorometer (Thermo Fisher Scientific, Waltham, MA, USA), and the purity and integrity of the DNA were assessed using 1% agarose gel electrophoresis.

Using VAHTS Universal Plus, a DNA Library Prep Kit for an Illumina NovaSeq 6000 (ND617, Vazyme, Nanjing, China), for library construction, we followed the instructions for operation. After the DNA samples passed quality control, the DNA was randomly fragmented using ultrasonication. The DNA fragments were then subjected to end repair, 3′ end A-tailing, sequencing adapter ligation, purification, and PCR amplification to construct the sequencing library. Once the library passed quality control, sequencing was performed on the Illumina NovaSeq 6000 platform (San Diego, CA, USA). The raw sequencing data were assessed and filtered for quality using Cutadapt software (V1.18) [22] and Trimmomatic software (V0.40) [23]. The main steps for data filtering were as follows: (1) If the length of the reads after filtering was less than 100bp, they were filtered out in pairs. If it was greater than 100bp, these reads could be used for subsequent analysis and were retained. (2) If the N content of a read exceeded 10%, paired reads were filtered out. (3) If 50% of the base mass values in a read was below Q20, paired reads were filtered out. (4) The processed double terminal reads were aligned with the reference genome to detect the actual size of the sequencing fragment obtained after DNA fragmentation of the sample, i.e., the insert size. The length distribution of inserted fragments follows a normal distribution, indicating that the construction of the sequencing data library is normal. In December 2020, Ma et al. (https://db.cngb.org/search/assembly/CNA0017758/ (accessed on 2 December 2020), MER) successfully assembled high-quality reference genomes of passion fruit for the first time, with sizes of 1.28 Gb, each consisting of 9 chromosomes. We evaluated the assembly quality of two genomes and selected the passion fruit genome assembled by MER as a reference. Using the passion fruit genome as a reference, we employed BWA software [24] to align the clean reads to the reference genome, generating SAM format alignment results. SAMtools software (version 1.3.1) was then used to convert the SAM files into BAM format. Subsequently, the Picard tool (version 1.91) (http://sourceforge.net/projects/picard/ (accessed on 11 October 2021)) was used to sort the reads in the BAM files using the SortSam function. The final BAM files were utilized for coverage and depth statistics, as well as for variant calling. Next, the HaplotypeCaller module from the GATK (version 3.7) software package [25] was used to generate gvcf files for each sample, followed by SNP detection across all samples using the GenotypeGVCFs module. The main SNP detecting process was as follows: (1) For the results obtained from BWA alignment, Picard’s Mark Duplicate toolkit was used to remove PCR duplications. (2) GATK was employed for insertion-deletion (InDel) realignment, specifically realigning around insertion-deletion regions to correct errors in alignment results caused by InDels. (3) GATK was used for base quality score recalibration (Base Recalibration) to correct base quality values and detect variants, including SNPs and InDels (1–5 bp). (4) Strict filtering was applied to SNPs and InDels: SNP clusters were filtered if there were 2 SNPs within 5 bp; SNPs near InDels were filtered if there were SNPs within 5 bp of an InDel; and adjacent InDels were filtered (InDels less than 10 bp apart).

### 2.4. Linkage Map Construction

Based on the resequencing results, SNP genotyping and filtering were performed on the parents and 158 RILs. Following the method published by Huang et al. [26], a sliding window approach was used, where each window contained 15 SNPs, and the step size was 1 SNP along the chromosome. If 13 or more SNPs in the window were genotyped as “aa”, the window was classified as “aa”. Similarly, if 13 or more SNPs were genotyped as “bb”, the window was classified as “bb.” In other cases, the genotype was filled and corrected as “ab” [27]. After completing tag filling and correction, bin partitioning was performed based on the recombination of offspring. Each sample was arranged neatly according to the physical position of the chromosome. When there was a typing transition in any sample, it was considered that a recombination breakpoint had occurred. The SNPs between recombination breakpoints were marked as a bin, and no recombination events are considered to have occurred within the bin. Finally, the bin was used as a marker for constructing the genetic map.

HighMap software [28] was used for linkage grouping, marker ordering, genotype correction, and map evaluation. (1) The recombination rate and LOD value between markers were estimated using two-point analysis, and the markers were divided into 24 linkage groups based on the LOD value. (2) The improved Gibbs sampling method, spatial sampling method, and simulated annealing algorithm were combined and applied to the iterative process of bin marker mapping. Based on the contribution of parents to the genotype, the SMOOTHI method [29] was used to correct the marker typing. Missing genotypes were estimated using the k-nearest neighbor algorithm [26]. Using the nearest neighbor algorithm, the partially separated markers were added to the genetic map. (3) By following a cycle of correcting one row of graphs, a high-quality genetic map was ultimately obtained.

### 2.5. QTL Analysis and Candidate Gene Annotation

QTL mapping of stem rot disease in passion fruit was performed using the interval mapping method in MapQTL 6 (https://www.kyazma.nl/index.php/MapQTL/Updates/ (accessed on 14 January 2025)), and a permutation test (PT) was conducted 1000 times to set the threshold value. The IM algorithm in the MapQTL software was used for QTL mapping. When the LOD score at a certain position exceeded the threshold, we would consider a QTL present at that location. At a 5% significance level, a critical value of 2.5 was used, meaning that an LOD score of ≥2.5 was taken as the threshold for determining the existence of a QTL. The phenotypic variance explained (PVE) was defined as: PVE = (1–10^−2×LOD/N^) × 100, where N is the number of individuals (N = 158) and LOD is the peak LOD value.

The candidate genes were annotated on the passion fruit genome (https://db.cngb.org/search/assembly/CNA0017758/ (accessed on 20 October 2021)). Using the physical position corresponding to the mapped interval, the candidate genes were screened through the annotation function of the passion fruit genome database.

### 2.6. RNA Sequencing

In the previous study, we artificially inoculated *Fusarium solani* on Huangjinguao and Ziguo 7, and then collected leaves at 0 h, 24 h, and 48 h. Total RNA was extracted using the TRIzol^®^ Reagent kit (Invitrogen, Carlsbad, CA, USA), following the specific protocol outlined in the user manual. mRNA was enriched using oligo (dT), followed by mRNA fragmentation, cDNA synthesis, and adaptor ligation, following the previously published protocol [30]. In this study, transcriptome sequencing of passion fruit was conducted using the Illumina NovaSeq 6000 sequencing platform (Illumina, USA). An Illumina PE library was constructed for 2 × 150 bp sequencing, and fastp software was used to perform quality control on the obtained sequencing data, including: (1) assessing base error rate distribution statistics; (2) evaluating base content distribution. The original data after quality control, the clean data (reads), were compared with the reference genome using Hisat2 software (Version 2.1.0). The reference genome source is https://db.cngb.org/search/assembly/CNA0017758/ (9 October 2022). Subsequently, mapped reads were obtained, and RSeQC-2.3.6 software was used to evaluate the quality of the transcriptome sequencing comparison results, including assessing sequencing saturation, gene coverage, distribution of reads in different regions of the reference genome, and the distribution of reads in different chromosomes. String Tie software (v12.0) was used to splice each sample and finally merge them together.

The overall expression levels of genes/transcripts were quantitatively analyzed using the RSEM software (Version 1.3.3) (http://deweylab.github.io/RSEM/ (accessed on 11 October 2022)). This analysis aimed to facilitate subsequent examination of differential gene/transcript expression between different samples. The quantitative metric used was TPM (transcripts per million). After obtaining read counts for gene expression analysis, DESeq2 was employed for differential expression analysis between samples or groups in the multi-sample project. Differentially expressed genes/transcripts were identified with the following parameters: *p* adjust < 0.05 and |log2FC| ≥ 1.

### 2.7. RT-qPCR

We planted 10 individuals each of HG and ZG7 in the greenhouse, taking care to isolate them from the outside world. We selected healthy plants for artificial inoculation of *Fusarium solani* during the 6–8 leaf stage. The specific method was to collect stem rot samples and isolate the pathogenic bacteria, place them on potato glucose agar medium (PDA) for isolation and purification, and the main pathogenic bacteria was *Fusarium oxysporum*. The purified and stored pathogenic bacteria were activated and inoculated onto PDA medium. After incubation at 28 ° C for 5 days, a 1000 μL pipette tip was used to punch holes to obtain mycelial blocks. Then, the in vitro inoculation method was used for disease resistance identification. The stem base was manually punctured, and the prepared mycelium blocks were picked up to the puncture site, with the front of the mycelium tightly attached to the wound. Referring to a previous study [4], samples were taken at the lesion site 72 h after inoculation with *Fusarium solani*, and three biological replicates were carried out for each sample, quickly frozen in liquid nitrogen, and transferred to a −80 °C refrigerator for storage.

Total RNA was extracted using the TRIzol^®^ Reagent kit (Invitrogen, California, USA), following the manufacturer’s instructions. We referred to previous research and selected *EF1* [30] and *EF-1α-2* [4] as reference genes. RT-qPCR was employed to validate the candidate genes and primers listed in Supplementary Table S7. All RT-qPCR assays were carried out in 96-well plates using a qTOWER 2.2 Quantitative Real-Time PCR Thermal Cycler (Analytik, Jena, Germany). The reaction system included: 10 µL of 2 × TransStart SYBR Green Master Mix (Vazyme, Nanjing, China), 1 µL of each primer, 1 µL of template cDNA, complemented by ddH2O to 20 µL. The cycle program for product amplification was as follows: 94 °C for 5 min followed by 40 cycles of 94 °C for 30 s (denaturation), 55 °C for 30 s (annealing), and 72 °C for 30 s (extension). Triplicates were carried out for each sample. When the reaction was completed, the melting curve was analyzed and specificity of the product was determined. The relative gene expression level was calculated by reference to the 2^−ΔΔCt^ method [31].

### 2.8. Statistical Analysis

We used OriginPro 2019b 32-bit software (https://www.originlab.com/) to draw graphics using the histogram drawing module. CorelDRAW X8 (https://www.coreldraw.com/cn/) software was used for data visualization and figure generation. The values are shown as mean ± SD. Statistical significance was determined in Excel (2019) using 2-tailed Student’s *t*-tests for pairwise comparisons (* *p* < 0.05, ** *p* < 0.01, and *** *p* < 0.001). All primers were designed using Primer Premier 5 (http://www.premierbiosoft.com/).

## 3. Results

### 3.1. Genotyping-by-Resequencing

Genotyping of the two parents, HG and ZG7, along with 158 BC_1_F_1_ individuals, was performed using the Illumina NovaSeq 6000 sequencing platform. HG and ZG7 obtained 41.9 Gb and 42.0 Gb of clean data, respectively, with an average sequencing depth of 30×; 158 offspring obtained 868.3 Gb of clean data with an average sequencing depth of 4×. The percentage of bases with sequencing quality values greater than or equal to 30 for parents and offspring is 93.95% (Table S1).

Based on the passion fruit reference genome, BWA software (Version 0.7.17) was used to realign the clean reads obtained from the resequencing of the two parents and 158 progenies to the reference genome. HG and ZG7 obtained 28,3754,952 and 282,230,426 clean reads, with the percentage of clean reads mapped in the passion fruit reference genome accounting for 98.63% and 98.75% of all clean reads, respectively. The offspring obtained an average of 36, 970,400 clean reads, with a mapped rate of 98.18% (Table S2).The average alignment efficiency for all samples was above 90%, indicating that the sequencing quality was normal.

The reads aligned to the reference genome were analyzed to determine the percentage of sequencing bases that covered the passion fruit genome, and the coverage depth was calculated. The average genome coverage depth for the parent samples was over 20×, with genome coverage exceeding 90% (at least 1× coverage). For the progeny samples, the average coverage depth was 3.92×, with coverage exceeding 74.87% (at least 1× coverage) (Table S3).

### 3.2. SNP Detection

A total of 5,849,756 SNPs were detected between the two parents, including 4,096,174 transitions and 1,753,582 transversions. ZG7 had 3,566,274 heterozygous SNPs and 2,283,482 homozygous SNPs, while HG had 946,585 heterozygous SNPs and 4,903,171 homozygous SNPs (Table S4). The higher the number of homozygous SNPs, the greater the difference between the sample and the reference genome. Conversely, the higher the number of heterozygous SNPs, the higher the degree of heterozygosity, which relates to the specific characteristics of the selected materials.

### 3.3. Genetic Linkage Map

Using the 1,180,406 SNPs obtained, we applied a sliding window approach with 15 SNPs per window and a step size of 1 SNP across the chromosomes. If the number of SNPs with the genotype “aa” within the window was greater than or equal to 13, the window was classified as “aa”; if the number of SNPs with the genotype “bb” was greater than or equal to 13, the window was classified as “bb”. In other cases, “ab” was used for genotype imputation and correction. After completing the imputation and correction of markers, bin segmentation was performed based on the recombination events in the progeny. Ultimately, 4206 valid bin markers were obtained on the passion fruit genome.

Using 4206 bin markers, a graphical genotype analysis of the 158 progenies was performed. In most lines, there are chromosomes without recombination, entirely derived from a single parental genome. Some chromosome segments in certain lines are heterozygous, which may be due to incomplete or erroneous repair after chromosome crossover.

**Figure 1:**
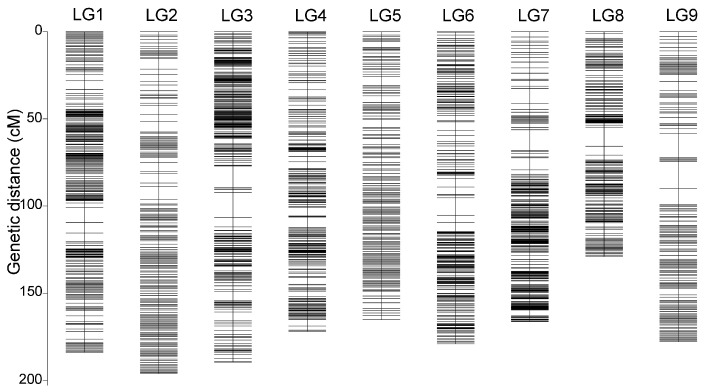
Genetic lengths and marker distribution of nine linkage groups in the genetic map of passion fruit. The x-axis represents the linkage groups; the y-axis represents the genetic distance.

Using the known information, the bins were divided into nine linkage groups. For each linkage group, HighMap software (Version 2021.11) [28] was employed to analyze the linear arrangement of markers and estimate the genetic distance between adjacent markers. The final genetic map covered a total length of 1559.03 cM (Figure 1).

The nine linkage groups of passion fruit contained a total of 4206 bin markers, ranging from 308 to 956 markers per group. The total genetic distance was 1559.03 cM, with an average genetic distance of 0.37 cM between bin markers. The largest gap between markers, 15.45 cM, was located on LG9. Detailed information is presented in Table 1.

### 3.4. Linkage Assessment and Collinearity Analysis

The genetic map is essentially a result of multipoint recombination analysis, where the smaller the genetic distance between markers, the lower the recombination rate. By analyzing the recombination relationships between markers and their neighboring markers, potential mapping errors or problematic markers can be identified. Supplementary Figure S1 presents the recombination heatmap of the markers, showing the linkage relationships across all linkage groups.

**Figure 2:**
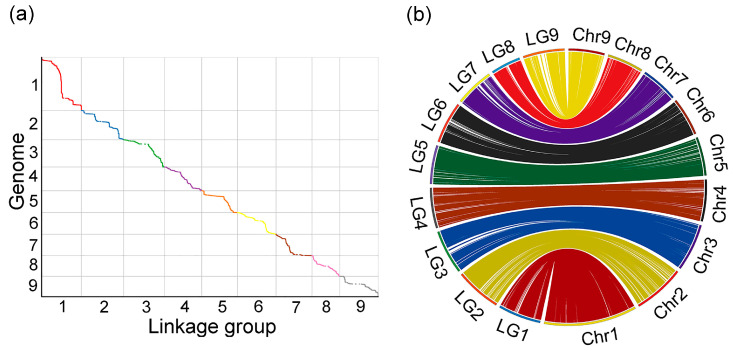
Collinearity between genetic and reference genome in passion fruit. (**a**) The relationship between linkage groups and genomes. The x-axis represents genetic distance; the y-axis represents physical distance. (**b**) Visualization of collinearity.

Next, a collinearity analysis was performed by using the position of the markers on the genome and the genetic map. This analysis helps to assess the accuracy of the genetic map compared to the reference genome. The collinearity between the genetic map and the physical genome is depicted in the figure below. To further quantify the relationship between each linkage group, Spearman’s correlation coefficient was calculated for each linkage group. The closer the Spearman coefficient is to 1, the better the collinearity between the genetic map and the physical genome. The Spearman correlation for each linkage group with the reference genome is shown in Figure 2.

### 3.5. Analysis of Passion Fruit Stem Rot

**Figure 3:**
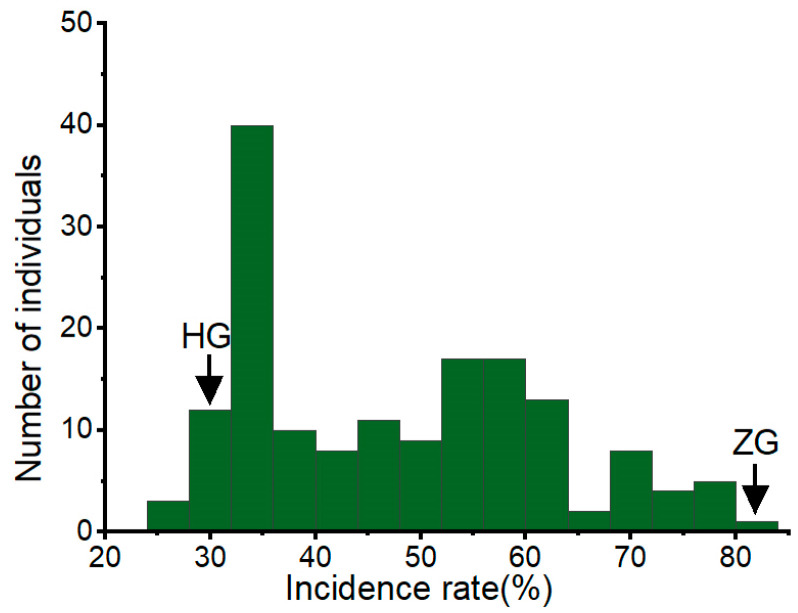
Distribution of stem rot disease resistance in the passion fruit BC_1_F_1_ population. The x-axis shows the ranges of phenotypic traits and the y-axis represents the number of individuals in the BC_1_F_1_ population.

The identification results showed that the incidence rates of HG and ZG7 were 29.7% and 81.2%, respectively. The incidence rate of stem rot in the BC_1_F_1_ population was continuously distributed, showing a quantitative genetic model (Figure 3). The minimum incidence rate in 158 individuals was 25.4%, and the maximum was 81.2%. The *t*-test indicated a significant difference between the two extreme values.

### 3.6. Mapping of Resistance Loci for Stem Rot and Analysis of Candidate Genes

**Figure 4:**
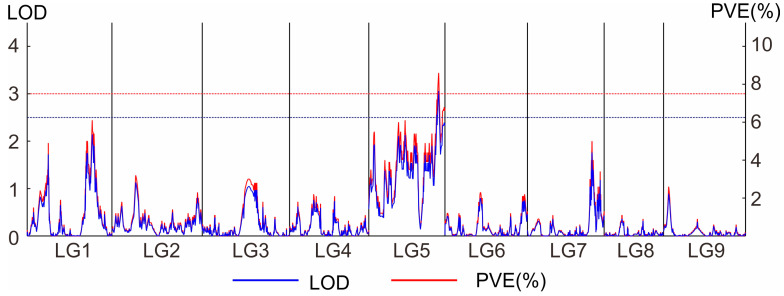
QTL mapping for stem rot disease resistance on linkage group 5 in passion fruit. The x-axis represents the linkage groups. LOD: the logarithm of odds score; PVE: the percentage of the phenotypic variance explained by an individual QTL. Blue line, LOD = 2.5; red line, LOD = 3.0.

The MapQTL interval mapping method was used to locate the trait, and the threshold was set by 1000 PT tests. A total of one QTL was detected for passion fruit stem rot resistance, located at 145.878–152.951 cM on linkage group 5, with phenotypic variance explained (PVE) of 8.6% (Figure 4). Subsequently, we named this gene the quantitative trait locus for passion fruit stem rot disease resistance on chromosome 5 (*qPSR5*).

Based on the analysis results, we aligned the bin markers to the passion fruit genome within the 113,377,860–114,811,870 bp region, which contains 33 candidate genes (Supplementary Table S5). Among them, *ZX.05G0020700*, *ZX.05G0020740*, *ZX.05G0020790*, and *ZX.05G0020880* encode NAC domain proteins. Previous studies have shown that NAC transcription factors enhance plant responses to biotic stress by regulating the expression of disease resistance-related genes [32,33,34,35]. *ZX.05G0020810* is also associated with increased disease resistance, encoding a zinc ribbon domain-containing protein. In the previous studies, we used RNA-seq to identify differentially expressed genes (DEGs) in response to *Fusarium solani* infection in both HG and ZG7, identifying a total of 6801 DEGs [12] (Supplementary Table S6). Within the candidate region for the stem rot resistance gene *qPSR5*, there are five DEGs (Table S6). One of these, *ZX.05G0020920*, encodes a WAT1-related protein, and the *WAT1* gene (*At01g75500*) in *Arabidopsis* has been shown to mediate resistance to pathogens [36,37] (Table 2).

**Figure 5:**
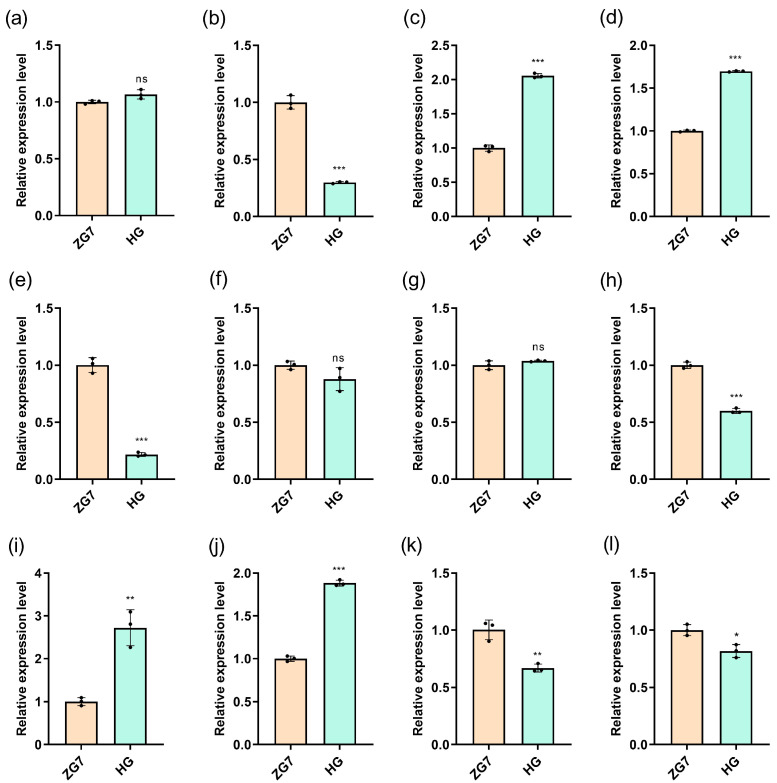
Relative expression level of candidate genes between Huangguoyuanshengzhong (HG) and Ziguo 7 (ZG7). (**a**–**f**) represent *ZX.05G0020700*, *ZX.05G0020740*, *ZX.05G0020760*, *ZX.05G0020810*, *ZX.05G0020830*, and *ZX.05G0020920*, and *EF1* as the reference gene; (**g**–**l**) represent *ZX.05G0020700*, *ZX.05G0020740*, *ZX.05G0020760*, *ZX.05G0020810*, *ZX.05G0020830*, and *ZX.05G0020920,* and *EF-1 α-2* as the reference gene. The x-axis represents the expression level of genes; the y-axis represents the variety of passion fruit. Data are represented as mean ± SD. * *p* < 0.05, ** *p* < 0.01, *** *p* < 0.0001, ns indicates no significance, with Student’s *t*-test.

Using *EF1* and *EF-1 α-2* as a reference gene, we performed RT-qPCR to detect the expression levels of *ZX.05G0020700*, *ZX.05G0020740*, *ZX.05G0020760*, *ZX.05G0020810*, *ZX.05G0020830*, and *ZX.05G0020920* in both HG and ZG7 (Figure 5). The results indicated that the expression levels of *ZX.05G0020740* and *ZX.05G0020810* were significantly different in HG and ZG7, while the expression level of *ZX.05G0020700* had no significance.

## 4. Discussion

The passion fruit has an aromatic aroma and is rich in sugars, vitamins, and mineral elements such as calcium, iron, and zinc [38,39]. It has extremely high nutritional value and is deeply loved by consumers. However, the occurrence and spread of stem rot caused by fungi such as *Fusarium oxysporum*, *Fusarium solani*, and *Phytophthora nicotianae* in Southern China pose a serious threat to the production of passion fruit. The use of molecular breeding technology to cultivate new varieties resistant to diseases is the most effective and economical method for disease prevention and control [10], but there are currently no reports on stem rot resistance genes/QTLs in passion fruit both domestically and internationally. Therefore, it is of great significance to explore the resistance genes of stem rot in passion fruit and cultivate varieties resistant to stem rot.

Genetic maps are commonly used for quantitative trait locus mapping, in order to obtain genome fragments that are linked to the target trait. In this study, we identified a QTL *qPSR5* by passion fruit high-density genetic mapping, but its PVE is low. Previous studies have shown that in a population without singular separation, the genetic variance of a QTL only depends on the genetic effects of the QTL, and QTLs with larger effects also have higher PVE. If there is a singular separation, the genetic variance of a QTL depends not only on the genetic effects of the QTL, but also on genotype frequency. QTLs with larger effects may not necessarily have higher PVE [40]. Wang et al. used a genetic map of oysters (*Crassostrea gigas* × *Crassostrea angulata*) to map 27 growth-related loci, with PVE ranging from 4.2% to 7.3% [41]. Wang et al. identified 22 yield-related blade length and width loci using high-density genetic maps of *Saccharina japonica*, but 17 loci had PVE values less than 10% [42]. Jiang et al. identified 11 QTLs of resistance to *Aspergillus flavus* infection using a high-density genetic map of peanuts, with PVE ranging from 5.03% to 10.87%. Only three loci had a PVE greater than 10% [43]. Liu et al. used a genetic map of common wheat to identify four stable QTLs for powdery mildew resistance, *QPm.caas-2AS*, *QPm.caas-4AS*, *QPm.caas-4BL*, and *QPm.caas-6BS.* In different environments, the PVE of *QPM.caas-2AS* is 5.6–7.6%, the PVE of *QPM.caas-4AS* is 7.5–8.3%, and the PVE of *QPM.caas-6BS* is 7.6–9.6%, and their genetic effects were validated in a natural population including 100 cultivars [44]. Therefore, a low PVE in QTL analysis is also a common occurrence.

*qPSR5* was mapped to the 1.43 Mb interval on chromosome 5, which contains 33 candidate genes. Based on RNA-seq and RT-qPCR analyses, we believe that *ZX.05G0020700*, *ZX.05G0020740*, *ZX.05G0020760*, *ZX.05G0020810*, *ZX.05G0020830,* and *ZX.05G0020920* are the most likely candidate genes. In a previous study, we found that genes related to reactive oxygen species (ROS), lignin biosynthesis, and leucine-rich repeat domain proteins play a crucial role in passion fruit resistance to stem rot disease [12]. In this study, both *ZX.05G0020700* and *ZX.05G0020740* are NAC transcription factors. NAC transcription factors in plants not only regulate the growth of secondary cell walls [45,46,47,48] but also participate in responses to biotic and abiotic stresses [49]. Research has shown that lignin biosynthesis plays a diversified role in plant disease resistance [50,51], with NAC transcription factors regulating this process. For example, in rice, OsNAC5 can directly activate the expression of *OsCCR10*, which is involved in the biosynthesis of H- and G-lignin, thereby regulating drought tolerance by controlling lignin accumulation [52]. OsNAC028 is involved in lignin synthesis and positively regulates resistance to rice sheath blight [53]. OsNAC29/31 are top-tier transcription factors controlling secondary wall formation and activating Os*MYB61*, which in turn stimulates the expression of secondary wall cellulose synthesis genes [54]. OsNAC045 reduces stress-induced root growth inhibition by enhancing lignin synthesis in roots and reducing ROS accumulation in response to cold and salt stresses [55]. In wheat, TaNAC032 regulates lignin biosynthesis and enhances resistance to *Fusarium* head blight [33]. NAC transcription factors also regulate disease resistance genes, enhancing plant immunity. For instance, OsMNAC3 activates negative immune regulators such as *OsINO80*, *OsJAZ10*, and *OsJAZ11*, which negatively regulate resistance to rice blast and bacterial blight [35]. After *Fusarium kyushuense* infection, 15 *NAC* genes showed significantly different expression levels in yellow and purple passion fruit, with *PeNAC001*, *PeNAC003*, *PeNAC028*, *PeNAC033*, *PeNAC057*, *PeNAC058*, *PeNAC063*, and *PeNAC077* suspected to play key roles in *Fusarium kyushuense* resistance [56]. Moreover, PeNAC transcription factors may regulate the *PeKCS* gene, improving wax and very-long-chain fatty acid biosynthesis, and improvement of passion fruit resistance under *Fusarium kyushuense* and drought stress conditions [57]. In this study, *ZX.05G0020700* (*PeNAC068*) and *ZX.05G0020740* (*PeNAC069*) exhibited significant expression changes after *Fusarium solani* infection in HG and ZG7, suggesting these genes may play important roles in passion fruit resistance to stem rot disease.

*WAT1* (*Walls Are Thin 1*) is an *Arabidopsis* homolog of *Medicago truncatula NODULIN21*, essential for secondary wall formation in fibers [58]. The secondary cell wall (SCW) is a critical part of the plant cell wall, located inside the primary wall and gradually formed and thickened during cell growth and development. SCWs are composed primarily of complex polymers like cellulose, lignin, and hemicellulose. These components and structures provide SCWs with many important functions, such as mechanical support, water and nutrient transport, and resistance against pathogen invasion [59]. Denancé found that *Arabidopsis* wat1 (walls are thin1) mediates resistance to the bacterial vascular pathogen *Ralstonia solanacearum* [36]. Koseoglou et al. showed that inactivation of *WAT1* in tomato reduced susceptibility to *Clavibacter michiganensis* by downregulating bacterial virulence factors [60]. In this study, significant differences in *NAC* and *WAT1* gene expression were observed between HG and ZG7, and previous research has demonstrated that NAC transcription factors regulate the growth of plant secondary cell walls [45,46,47,48]. Therefore, it is likely that NAC transcription factors and *WAT1* genes jointly contribute to the regulation of passion fruit stem rot disease resistance.

## 5. Conclusions

In this study, we constructed a high-density genetic linkage map for passion fruit. Using this genetic map, a QTL *qPSR5* for resistance to stem rot disease was mapped on chromosome 5 in passion fruit. Candidate gene analyses suggest that NAC transcription factors may regulated resistance to stem rot in passion fruit. Moreover, ENHANCED DISEASE RESISTANCE 4 protein may also play an important role in the resistance to stem rot disease in passion fruit. Next, we will further fine-map *qPSR5* and validate its function using transgenic technology.

## Figures and Tables

**Table 1:** Statistical analysis of genetic map information of passion fruit.

Linkage Group	Total Bin Markers	Total Distance (cM)	Average Distance (cM)	Max Gap (cM)	Gaps < 5 cM (%)
LG1	956	183.92	0.19	8.65	98.12%
LG2	371	196.03	0.53	7.5	98.38%
LG3	509	189.5	0.37	14.49	99.02%
LG4	308	171.98	0.56	5.95	99.67%
LG5	378	165.26	0.44	5.34	99.73%
LG6	407	179.08	0.44	10.01	99.51%
LG7	537	166.35	0.31	11.9	99.44%
LG8	364	129.04	0.36	10.91	99.45%
LG9	376	177.87	0.47	15.45	98.93%
Total	4206	1559.03	0.37	15.45	98.12%

**Table 2:** Candidate genes and annotation information for stem rot disease resistance in passion fruit.

Gene	Annotation
*ZX.05G0020700*	NAC domain-containing protein 14
*ZX.05G0020740*	NAC domain-containing protein 91
*ZX.05G0020760*	DNA/RNA polymerases superfamily protein
*ZX.05G0020810*	Protein ENHANCED DISEASE RESISTANCE 4
*ZX.05G0020830*	B3 domain-containing transcription factor
*ZX.05G0020920*	WAT1-related protein

## Data Availability

The resequencing data are deposited in the NCBI (SRA) database with the accession number PRJNA1193301 (SRA nos. from SRR31644989 to SRR31645148). The RNA-seq data that support the findings of this study have been deposited in the National Center for Biotechnology Information (NCBI) Sequence Read Archive (SRA) with the accession code PRJNA1102446 (SRA nos. from SRR28748077 to SRR28748094).

## Supplementary Materials

The following supporting information can be downloaded at: https://www.mdpi.com/article/10.3390/genes16010096/s1, Figure S1. Marker linkage relationship diagram of nine linkage groups in passion fruit. Each row and column are arranged in the order of the graph as markers, with each small square representing the recombination rate between two markers. The change in color from yellow to red to purple represents the variation in recombination rate from small to large. The closer the distance, the lower the recombination rate of the marker, and the closer the color is to yellow. The farther the distance, the higher the recombination rate of the marker, and the closer it is to purple. Table S1. Statistics of sequencing data for ZG7, HG and 158 individuals. Table S2. Comparison of ZG7, HG, and 158 incividuals to the passion fruit genome. Table S3. Depth and coverage of sequencing data for ZG7, HG and 158 individuals. Table S4. The decetation of SNPs between ZG7 and HG. Table S5. Candidate genes for resistance to stem rot disease in passion fruit. Table S6. DEGs during three stages of Fusarium solani infection between HG and ZG7. Table S7. Primers used in this study.

## References

[1] Aiello D., Fiorenza A., Leonardi G.R., Vitale A., Polizzi G. (2021). *Fusarium nirenbergiae* (*Fusarium oxysporum* species complex) causing the wilting of passion fruit in Italy. Plants.

[2] Wang C., Ye X., Ng T.B., Zhang W. (2021). Study on the biocontrol potential of antifungal peptides produced by *Bacillus velezensis* against *Fusarium solani* that infects the passion fruit *Passiflora edulis*. J. Agric. Food Chem..

[3] Liu Z.L., Zhou S., Huang Y., Yang L., Yan Y., Chen G., Sun J.M., Wu S., Chen X. (2021). First report of fruit rot caused by *Phytophthora nicotianae* on passion fruit in Guangxi province, China. Plant Dis..

[4] Wu Y., Tian Q., Huang W., Liu J., Xia X., Yang X., Mou H. (2020). Identification and evaluation of reference genes for quantitative real-time PCR analysis in *Passiflora edulis* under stem rot condition. Mol. Biol. Rep..

[5] Rooney-latham S., Blomquist C.L., Scheck H.J. (2011). First report of *Fusarium wilt* caused by *Fusarium oxysporum* f. sp. *passiflorae* on passion fruit in North America. Plant Dis..

[6] Bueno C.J., Fischer I.H., Rosa D.D., Firmino A.C., Harakava R., Oliveira C.G., Furtad E.L. (2013). *Fusarium solani* f.sp. *passiflorae*: A new *Forma specialis* causing collar rot in yellow passion fruit. Plant Pathol..

[7] Fischer I.H., Rezende J.M. (2008). Diseases of passion flower (*Passiflora* spp.). Pest. Technol..

[8] Thuranira M., Wasilwa L., Matiru V. (2011). Effect of soil management and *Trichoderma asperellum* on severity of passionfruit wilt disease. Acta Hortic..

[9] Chen Y.H., Lee P.C., Huang T.P. (2021). Biological control of collar rot on passion fruits via induction of apoptosis in the collar rot pathogen by Bacillus subtilis. Phytopathology.

[10] Nelson R., Wiesner-hanks T., Wisser R., Balint-kurti P. (2018). Navigating complexity to breed disease-resistant crops. Nat. Rev. Genet..

[11] Ribeiro R.M., Viana A.P., Santos E.A., Rodrigues D.L., Da Costa Preisigke S. (2019). Breeding passion fruit populations-review and perspectives. Funct. Plant Breed. J..

[12] Wu Y., Shi G., Zhou J., Tian Q., Liu J., Huang W., Xia X., Mou H., Yang X. (2025). Identification and validation of stem rot disease resistance genes in passion fruit (*Passiflora edulis*). Hortic. Sci..

[13] Yang X., Zhong S., Zhang Q., Ren Y., Sun C., Chen F. (2021). A loss-of-function of the dirigent gene *TaDIR-B1* improves resistance to *Fusarium crown* rot in wheat. Plant Biotechnol. J..

[14] Lv G., Zhang Y., Ma L., Yan X., Yuan M., Chen J., Cheng Y., Yang X., Qiao Q., Zhang L. (2023). A cell wall invertase modulates resistance to fusarium crown rot and sharp eyespot in common wheat. J. Integr. Plant Biol..

[15] Chen Y., Wang Y., Guan F., Long L., Wang Y., Li H., Deng M., Zhang Y., Pu Z., Li W. (2023). Comparative analysis of *Fusarium crown* rot resistance in synthetic hexaploid wheats and their parental genotypes. BMC Genomics.

[16] Liu C., He S., Chen J., Wang M., Li Z., Wei L., Chen Y., Du M., Liu D., Li C. (2024). A dual-subcellular localized β-glucosidase confers pathogen and insect resistance without a yield penalty in maize. Plant Biotechnol. J..

[17] Cao Y., Ma J., Han S., Hou M., Wei X., Zhang X., Zhang Z.J., Sun S., Ku L., Tang J. (2023). Single-cell RNA sequencing profiles reveal cell type-specific transcriptional regulation networks conditioning fungal invasion in maize roots. Plant. Biotechnol. J..

[18] Cao Y., Yan X., Ran S., Ralph J., Smith R.A., Chen X., Qu C., Li J., Liu L. (2022). Knockout of the lignin pathway gene *BnF5H* decreases the S/G lignin compositional ratio and improves *Sclerotinia sclerotiorum* resistance in *Brassica napus*. Plant Cell Environ..

[19] Li H., Chen A., Tang H., Luan M. (2024). Genetic mapping is used for gene mapping/QTL mapping to obtain genome fragments that are linked to the target trait. BMC Plant Biol..

[20] Wang W., Xu Z., Qian L., Hang S., Niu Y., Shen C., Wei Y., Liu B. (2024). Genetic mapping and validation of QTL controlling fruit diameter in cucumber. BMC Plant Biol..

[21] Tamari F., Hinkley C.S., Ramprashad N. (2013). A comparison of DNA extraction methods using *Petunia hybrida* tissues. J. Biomol. Tech..

[22] Martin M. (2011). CUTADAPT removes adapter sequences from high-throughput sequencing reads. EMBnet J..

[23] Bolger A.M., Lohse M., Usadel B. (2014). Trimmomatic: A flexible trimmer for Illumina sequence data. Bioinformatics.

[24] Li H., Durbin R. (2009). Fast and accurate short read alignment with burrows-wheeler transform. Bioinformatics.

[25] Mckenna A., Hanna M., Banks E., Sivachenko A., Cibulskis K., Kernytsky A., Garimella K., Altshuler D., Gabriel S., Daly M. (2010). The Genome Analysis Toolkit: A MapReduce framework for analyzing next-generation DNA sequencing data. Genome Res..

[26] Huang X., Feng Q., Qian Q. (2009). High-throughput genotyping by whole-genome resequencing. Genome Res..

[27] Van Os H., Stam P., Visser R.G., Vaneck H.J. (2005). SMOOTH: A statistical method for successful removal of genotyping errors from high-density genetic linkage data. Theor. Appl. Genet..

[28] Liu D., Ma C., Hong W., Huang L., Liu M., Liu H., Zeng H., Deng D., Xin H., Song J. (2014). Construction and analysis of high-density linkage map using high-throughput sequencing data. PLoS ONE.

[29] Wu Y., Huang W., Tian Q., Liu J., Xia X., Yang X., Mou H. (2021). Comparative transcriptomic analysis reveals the cold acclimation during chilling stress in sensitive and resistant passion fruit (*Passiflora edulis*) cultivars. PeerJ.

[30] Zhao M., Fan H., Tu Z., Cai G., Zhang L., Li A., Xu M. (2022). Stable reference gene selection for quantitative real-time PCR normalization in passion fruit (*Passiflora edulis* Sims.). Mol. Biol. Rep..

[31] Livak K.J., Schmittgen T.D. (2001). Analysis of relative gene expression data using real-time quantitative PCR and the 2^−ΔΔC_T_^ method. Methods.

[32] Liu Q., Yan S., Huang W., Yang J., Dong J., Zhang S., Zhao J., Yang T., Mao X., Zhu X. (2018). NAC transcription factor ONAC066 positively regulates disease resistance by suppressing the ABA signaling pathway in rice. Plant Mol. Biol..

[33] Soni N., Altartouri B., Hegde N., Duggavathi R., Nazarian-firouzabadi F., Kushalappa A.C. (2021). TaNAC032 transcription factor regulates lignin-biosynthetic genes to combat Fusarium head blight in wheat. Plant Sci..

[34] Chen C., Jost M., Outram M.A., Friendship D., Chen J., Wang A., Periyannan S., Bartoš J., Holušová K., Doležel J. (2023). A pathogen-induced putative NAC transcription factor mediates leaf rust resistance in barley. Nat. Commun..

[35] Wang H., Bi Y., Yan Y., Yuan X., Gao Y., Noman M., Li D., Song F. (2024). A NAC transcription factor MNAC3-centered regulatory network negatively modulates rice immunity against blast disease. J. Integr. Plant Biol..

[36] Denancé N., Ranocha P., Oria N., Barlet X., Rivière M.P., Yadeta K.A., Hoffmann L., Perreau F., Clément G., Maia-grondard A. (2013). Arabidopsis *wat1* (*walls are thin1*)-mediated resistance to the bacterial vascular pathogen, *Ralstonia solanacearum*, is accompanied by cross-regulation of salicylic acid and tryptophan metabolism. Plant J..

[37] Hanika K., Schipper D., Chinnappa S., Oortwijn M., Schouten H.J., Thomma B., Bai Y. (2021). Impairment of tomato *WAT1* enhances resistance to Vascular wilt fungi despite severe growth defects. Front. Plant Sci..

[38] Xia Z., Huang D., Zhang S., Wang W., Ma F., Wu B., Xu Y., Xu B., Chen D., Zou M. (2021). Chromosome-scale genome assembly provides insights into the evolution and flavor synthesis of passion fruit (*Passiflora edulis* Sims). Hortic. Res..

[39] Yu C., Wang P., Zhang S., Liu J., Cheng Y., Zhang S., Wu Z. (2024). Passionfruit genomic database (PGD): A comprehensive resource for passionfruit genomics. BMC Genomics.

[40] Li H.H., Zhang L.Y., Wang J.K. (2010). Analysis and answers to frequently asked questions in quantitative trait locus mapping. ACTA Agronomica Sinica.

[41] Wang J., Li L., Zhang G. (2016). A high-density SNP genetic linkage map and QTL analysis of growth-related traits in a hybrid family of oysters (*Crassostrea gigas* × *Crassostrea angulata*) using genotyping-by-sequencing. G3.

[42] Wang X., Chen Z., Li Q., Zhang J., Liu S., Duan D. (2018). High-density SNP-based QTL mapping and candidate gene screening for yield-related blade length and width in *Saccharina japonica* (Laminariales, Phaeophyta). Sci. Rep..

[43] Jiang Y., Luo H., Yu B., Ding Y., Kang Y., Huang L., Zhou X., Liu N., Chen W., Guo J. (2021). High-density genetic linkage map construction using whole-genome resequencing for mapping QTLs of resistance to *Aspergillus flavus* infection in peanut. Front. Plant Sci..

[44] Liu X., Zhang X., Meng X., Liu P., Lei M., Jin H., Wang Y., Jin Y., Cui G., Mu Z. (2024). Identification of genetic loci for powdery mildew resistance in common wheat. Front. Plant Sci..

[45] Yamaguchi M., Ohtani M., Mitsuda N., Kubo M., Ohme-takagi M., Fukuda H., Demura T. (2010). VND-INTERACTING2, a NAC domain transcription factor, negatively regulates xylem vessel formation in *Arabidopsis*. Plant Cell.

[46] Cao S., Wang Y., Gao Y., Xu R., Ma J., Xu Z., Shang-guan K., Zhang B., Zhou Y. (2023). The RLCK-VND6 module coordinates secondary cell wall formation and adaptive growth in rice. Mol. Plant.

[47] Cong L., Shi Y.K., Gao X.Y., Zhao X.F., Zhang H.Q., Zhou F.L., Zhang H.J., Ma B.Q., Zhai R., Yang C.Q. (2024). Transcription factor PbNAC71 regulates xylem and vessel development to control plant height. Plant Physiol..

[48] Taylor-teeples M., Lin L., De Lucas M., Turco G., Toal T.W., Gaudinier A., Young N.F., Trabucco G.M., Veling M.T., Lamothe R. (2015). An Arabidopsis gene regulatory network for secondary cell wall synthesis. Nature.

[49] Nakashima K., Takasaki H., Mizoi J., Shinozaki K., Yamaguchi-shinozaki K. (2012). NAC transcription factors in plant abiotic stress responses. Biochim. Biophys. Acta.

[50] Ma Q.H. (2024). Lignin biosynthesis and its diversified roles in disease resistance. Genes.

[51] Liu Q., Luo L., Zheng L. (2018). Lignins: Biosynthesis and biological functions in plants. Int. J. Mol. Sci..

[52] Bang S.W., Choi S., Jin X., Jung S.E., Choi J.W., Seo J.S., Kim J.K. (2022). Transcriptional activation of rice *CINNAMOYL*-*CoA REDUCTASE 10* by OsNAC5, contributes to drought tolerance by modulating lignin accumulation in roots. Plant Biotechnol. J..

[53] Yuan P., Yang S., Feng L., Chu J., Dong H., Sun J., Chen H., Li Z., Yamamoto N., Zheng A. (2023). Red-light receptor phytochrome B inhibits BZR1-NAC028-CAD8B signaling to negatively regulate rice resistance to sheath blight. Plant Cell Environ..

[54] Huang D., Wang S., Zhang B., Shang-guan K., Shi Y., Zhang D., Liu X., Wu K., Xu Z., Fu X. (2015). A gibberellin-mediated DELLA-NAC signaling cascade regulates cellulose synthesis in rice. Plant Cell.

[55] Yu S., Huang A., Li J., Gao L., Feng Y., Pemberton E., Chen C. (2018). OsNAC45 plays complex roles by mediating POD activity and the expression of development-related genes under various abiotic stresses in rice root. Plant Growth Regul..

[56] Yang Q., Li B., Rizwan H.M., Sun K., Zeng J., Shi M., Guo T., Chen F. (2022). Genome-wide identification and comprehensive analyses of NAC transcription factor gene family and expression analysis under *Fusarium kyushuense* and drought stress conditions in *Passiflora edulis*. Front. Plant. Sci..

[57] Rizwan H.M., Shaozhong F., Li X., Bilal A.M., Yousef A.F., Chenglong Y., Shi M., Jaber M., Anwar M., Hu S.Y. (2022). Genome-wide identification and expression profiling of *KCS* gene family in passion fruit (*Passiflora edulis*) under *Fusarium kyushuense* and drought stress conditions. Front. Plant. Sci..

[58] Ranocha P., Denancé N., Vanholme R., Freydier A., Martinez Y., Hoffmann L., Köhler L., Pouzet C., Renou J.P., Sundberg B. (2010). *Walls are thin 1* (*WAT1*), an Arabidopsis homolog of *Medicago truncatula* NODULIN21, is a tonoplast-localized protein required for secondary wall formation in fibers. Plant J..

[59] Rao X., Dixon R.A. (2018). Current models for transcriptional regulation of secondary cell wall biosynthesis in grasses. Front. Plant. Sci..

[60] Koseoglou E., Hanika K., Mohd N.M., Kohlen W., Van der Wolf J.M., Visser R., Bai Y. (2023). Inactivation of tomato WAT1 leads to reduced susceptibility to *Clavibacter michiganensis* through downregulation of bacterial virulence factors. Front. Plant. Sci..

