# Supplementary figures and images for: Construction of a High-Density Genetic Linkage Map and QTL Mapping for Stem Rot Resistance in Passion Fruit (Passiflora edulis Sims)
Source: Genes (Basel). 2025 Jan 17;16(1):96. doi: 10.3390/genes16010096 (PMC11765416; doi:10.3390/genes16010096)

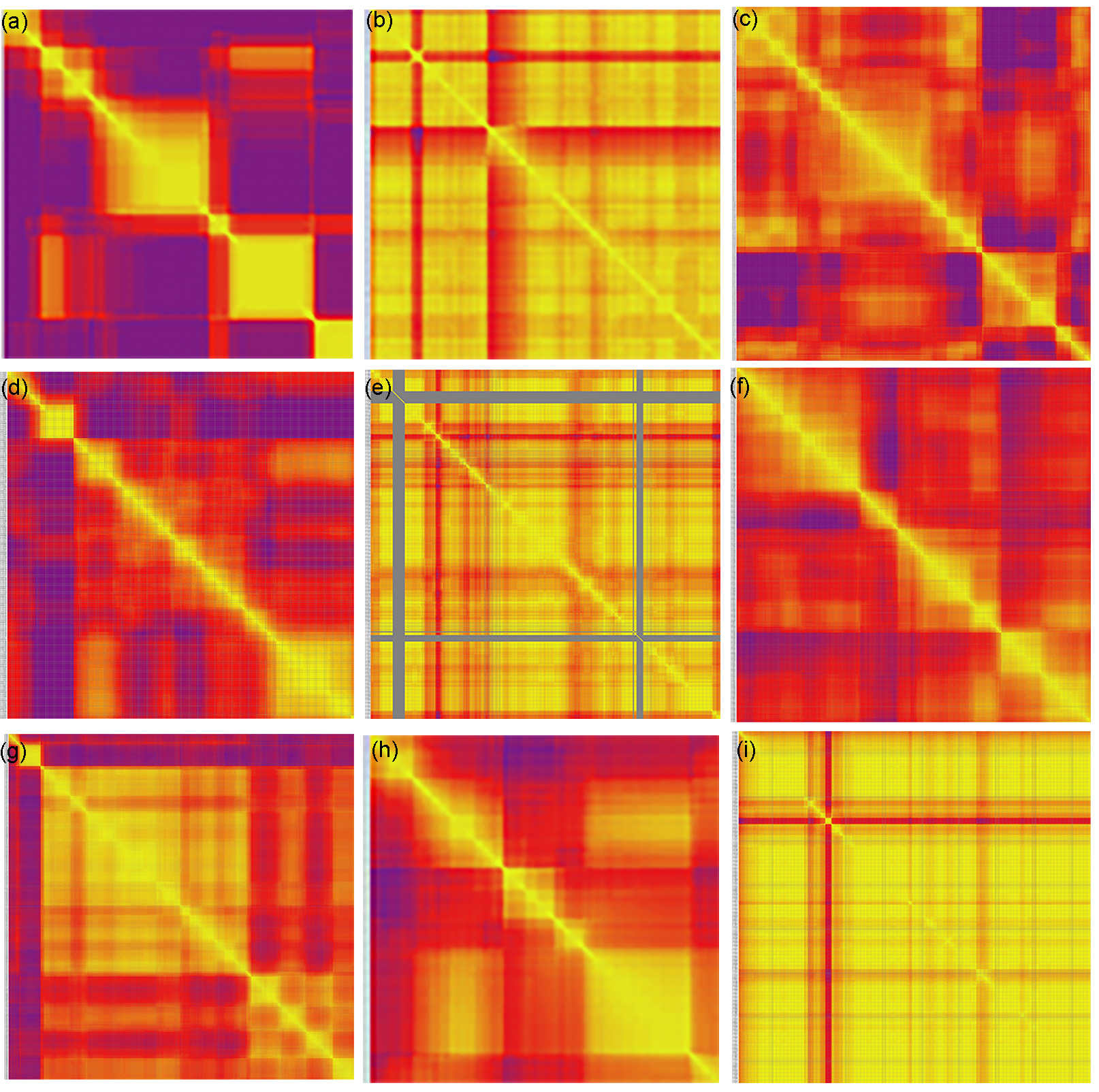

Supplement: Supplementary file 1 [file genes-16-00096-s001.zip › Figure S1.jpg]
